# Evaluating the process of partnership and research in global health: reflections from the STRIPE project

**DOI:** 10.1186/s12889-020-08591-y

**Published:** 2020-08-12

**Authors:** Anna Kalbarczyk, Aditi Rao, Yodi Mahendradhata, Piyusha Majumdar, Ellie Decker, Humayra Binte Anwar, Oluwaseun O. Akinyemi, Ahmad Omid Rahimi, Patrick Kayembe, Olakunle O. Alonge

**Affiliations:** 1 Department of International Health, Johns Hopkins Bloomberg School of Public Health, 615 N Wolfe St., Baltimore, MD USA; 2 Universitas Gadjah Mada Faculty of Medicine, Public Health, and Nursing, Yogyakarta, Indonesia; 3 Indian Institute of Health Management Research, Jaipur, India; 4 BRAC James P Grant School of Public Health, BRAC University, Dhaka, Bangladesh; 5 Department of Health Policy and Management, College of Medicine, University of Ibadan, Ibadan, Nigeria; 6 Global Innovations Consultancy Services, Kabul, Afghanistan; 7 University of Kinshasa School of Public Health, Kinshasa, Democratic Republic of the Congo

**Keywords:** Process evaluation, Partnership, Collaboration, Global health research, Expectations

## Abstract

**Background:**

Thoughtful and equitable engagement with international partners is key to successful research. STRIPE, a consortium of 8 academic and research institutions across the globe whose objective is to map, synthesize, and disseminate lessons learned from polio eradication, conducted a process evaluation of this partnership during the project’s first year which focused on knowledge mapping activities.

**Methods:**

The STRIPE consortium is led by Johns Hopkins University (JHU) in partnership with 6 universities and 1 research consultancy organization in polio free, at-risk, and endemic countries. In December 2018 JHU team members submitted written reflections on their experiences (*n* = 9). We held calls with each consortium member to solicit additional feedback (*n* = 7). To establish the partnership evaluation criteria we conducted preliminary analyses based on Blackstock’s framework evaluating participatory research. In April 2019, an in-person consortium meeting was held; one member from each institution was asked to join a process evaluation working group. This group reviewed the preliminary criteria, adding, subtracting, and combining as needed; the final evaluation criteria were applied to STRIPE’s research process and partnership and illustrative examples were provided.

**Results:**

Twelve evaluation criteria were defined and applied by each member of the consortium to their experience in the project. These included *access to resources*, *expectation setting*, *organizational context*, *external context*, *quality of information*, *relationship building*, *transparency*, *motivation*, *scheduling*, *adaptation*, *communication and engagement*, and *capacity building*. For each criteria members of the working group reflected on general and context-specific challenges and potential strategies to overcome them. Teams suggested providing more time for recruitment, training, reflection, pre-testing. and financing to alleviate resource constraints. Given the large scope of the project, competing priorities, and shifting demands the working group also suggested a minimum of one full-time project coordinator in each setting to manage resources.

**Conclusion:**

Successful management of multi-country, multicentered implementation research requires comprehensive communication tools (which to our knowledge do not exist yet or are not readily available), expectation setting, and institutional support. Capacity building activities that address human resource needs for both individuals and their institutions should be incorporated into early project planning.

## Background

The last three decades have seen substantive growth in partnerships between academic researchers and institutions in the global north and south [1], creating more efficient and collaborative approaches to generating knowledge [2], seeking to address global health disparities, and driving discoveries through analysis of large data sets [3].

As recognition of the importance of these partnerships in global health, particularly between academic institutions in the global north and south, the characteristics and evaluation criteria of successful partnerships have been developed. The Council for International Organizations of Medical Sciences (CIOMS) in collaboration with the World Health Organization (WHO), have recognized the critical importance of equitable partnerships by including collaboration guidelines in the International Ethical Guidelines for Health-related Research Involving Humans [4]. Specifically, guideline eight focuses on partnership and capacity-building; key aspects of guideline eight are collaborative partnerships, strengthening of research capacity, addressing conflicts of interest, education opportunities, and publication and data sharing practices [4]. The CIOMS guidelines echo the themes discussed by implementers and researchers. For example, through their participatory evaluation of collaboration across research centers Scarinci et al. found commitment from all stakeholders, participation, and meaningful engagement are necessary to successfully evaluate partnerships [5]. These partnership guidelines and metrics are necessary in global health given the historical challenges faced by global partnerships including unpredictable financing, low levels of trust, and lack of capacity building [6–8].

To understand lessons learned and best practices for the establishment and evaluation of partnerships studies have utilized evaluation frameworks with focus on demonstrating the effectiveness, equity or efficiency gains of those partnerships. Acknowledging that consortia, a subset of partnerships focused on a participatory approach to conducting research in multi-country settings, were established to combat health disparities between countries but often lacked methods to evaluate partnerships, Pratt and Hyder proposed a checklist to evaluate the governance of research consortia [9]. The equity-focused checklist is based on the components of shared health governance, including shared resources, responsibility, accountability, sovereignty, and justice; the authors suggest the checklist be used when developing consortia and during annual monitoring and evaluation activities [9]. Dankwa-Mullan and colleagues concentrate on health disparities research; they developed six general elements to guide teamwork for research involving many disciplines. These elements include the examination of the current institutional or societal culture, creation of an idea with a vision, development of a high-risk idea, identification of the changes that are needed with a focus on innovation, testing and implementation, and institutionalization of the idea. The authors retrospectively applied the six elements to research outcomes, though they challenge researchers to prospectively apply these concepts to guide future transdisciplinary research [10].

Blackstock, Kelly, and Horsey conducted a literature review to understand key components of participatory research and how to evaluate the success of participatory processes. This review informed the development of an evaluation framework which was then utilized to assess the participatory processes of a completed sustainability project [11]. Blackstock’s framework builds on existing work, including the Adaptive Integration of Research and Policy for Sustainable Development (AIRP-SD) evaluation framework for sustainable development, and similarly connects research outcomes, design, process, and context. The key difference is Blackstock et al. place the participatory approach, with an emphasis on co-construction of knowledge, in a central position. While the aforementioned projects did not complete evaluations while the project was ongoing, study authors recommended this for future work [9, 11].

Across these frameworks key concepts emerge, including the application of social justice principles to aspects of the partnership, understanding of the context in which the partnership is developed, and shared vision and goals [9–11]. Contextual factors such as insecurity and the political environment further exacerbate challenges to partnerships (e.g. untimely financing and distrust) [7]. Additionally, many of the guidelines to assess partnerships are developed in settings where low- and middle-income country (LMIC) researchers have little influence [8]. While creating and evaluating large-scale multi-country project partnerships is complex, evaluation of these relationships is essential to progress in the field of global health.

The *Synthesis and Translation of Research and Innovations from Polio Eradication (STRIPE)* project seeks to map, synthesize, and disseminate lessons learned from the global polio eradication effort via a mixed-methods, multi-country study. The project is a collaboration between the Johns Hopkins University (JHU), and seven academic and research country partners; Global Innovations Consultancy Services (Afghanistan), the James P Grant School of Public Health, BRAC University (Bangladesh), the University of Kinshasa School of Public Health (Democratic Republic of Congo), the School of Public Health, Addis Ababa University (Ethiopia), the Institute of Health Management Research (India), the Center for Tropical Medicine, Faculty of Medicine, Public Health and Nursing, Universitas Gadjah Mada (Indonesia), and the College of Medicine, University of Ibadan (Nigeria). These focus countries represent diverse epidemiological disease profiles; endemic, outbreak, at-risk, and polio-free. The first year of the project consisted of knowledge mapping activities which included a scoping review, large-scale global survey, key informant interviews (KIIs), and context mapping.

We conducted a process evaluation of this consortium partnership during the first year. This research seeks to prospectively evaluate the effectiveness and equity of partnerships in the process of conducting a large-scale, multi-country research project in collaboration with seven academic and research institutions as part of the STRIPE consortium. Specifically, we aimed to understand what worked well, what challenges emerged, and what solutions were developed and tried successfully within the first year of the five-year project. This process evaluation focuses on partnership relationships and consortium member experiences in addition to perceptions of the STRIPE project’s research activities.

## Methods

### Conceptual framework

**Fig. 1:**
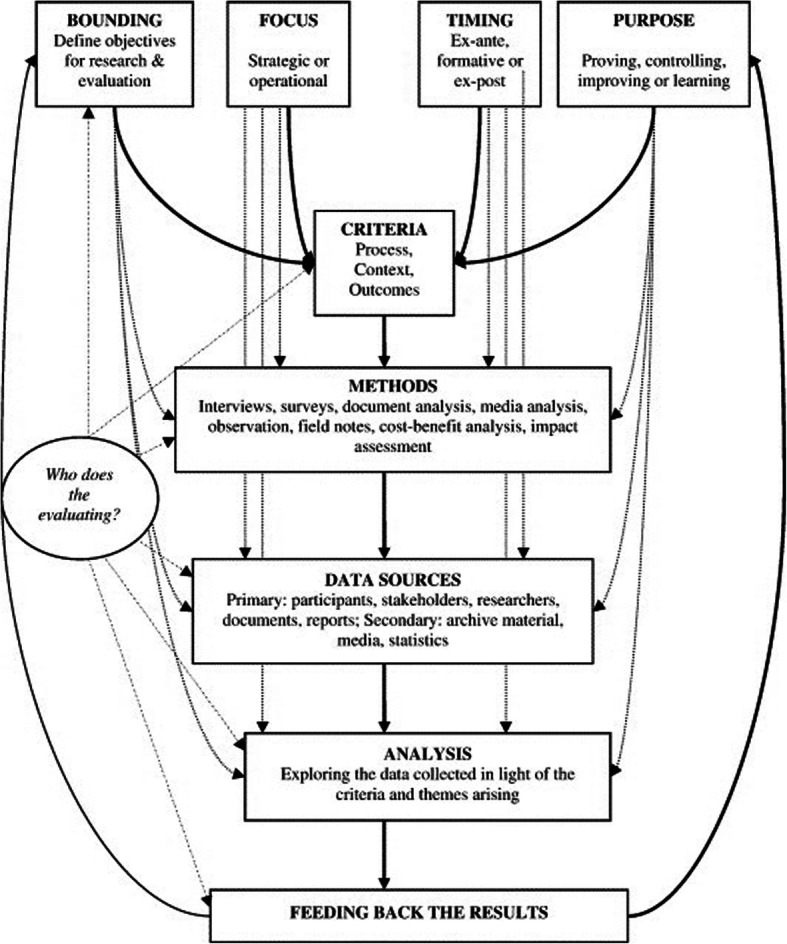
Blackstock et al. Framework. Permission for reproduction of this figure was provided via e-mail by the original author KL Blackstock on 28 October 2019

We utilized Blackstock et al.’s framework for evaluating participatory research and applied the constructs to the consortium’s activities and process in the first year of the project. Blackstock’s framework (see Fig. 1), built on substantive literature review, emphasizes the relationships between an evaluation’s purpose, the criteria selected by which the process will be evaluated, and the methods used to conduct the evaluation. Blackstock’s model was developed specific to the evaluation of participatory research and not to the practice of participatory evaluation (that is, evaluation conducted as a participatory process). This research sought to use the model to do both – to evaluate the consortium’s process in the first year and do so in collaboration with consortium members.

We began by defining the purpose, focus, bounding, and timing of the evaluation. This informed the selection of process, context, and outcome criteria. We used many of the original criteria constructs from the model, including access to resources, accountability, capacity, context, shared vision and goals, relationships, quality, and transparency. One modification of that model was that we defined our methods and data sources prior to the finalization of criteria as these criteria changed over time during the preliminary analysis process. The evaluators all worked collaboratively to both define the criteria and then apply them through the methodologies described below.

While consortium members did actively participate in many of the decision-making processes, it is important to note that the consortium relationship is contractual (sub-contracts issued from JHU to partner institutions) and therefore not entirely participatory.

### Data collection & analysis

At the end of the first year in December 2018, all JHU team members (*n* = 10) were asked to write a 2–3 page reflection on their experience with the project and partners. Specific questions included what went well, what changes occurred, what challenges were faced, what was done to address those challenges, etc. In total, 9 written reflections were submitted. Additionally, between December 2018–January 2019 the project manager initiated individual conference calls with each consortium member team. The purpose of these calls was two-fold: 1) connect on technical aspects of the remaining work, and 2) provide space for consortium team members to reflect on the past year. Notes were taken for each call. Preliminary analyses were conducted of the written reflections and notes; themes around the partnership and research processes were mapped to the Blackstock framework where applicable and new themes emerged creating unique evaluation criteria.

In April 2019 a 3-day consortium-wide meeting was held in Baltimore MD, USA, bringing together the primary and co-investigators of each consortium institution in addition to the full JHU team and representatives from the Bill & Melinda Gates Foundation. On the final day of the meeting, one representative from each country team was asked to join a process evaluation working group for a 90-min reflection session. The purpose of the process evaluation working group is to contribute to the consortium’s evaluation through a reflection on the partnership and processes for the first year of the project, share experiences, and provide recommendations. Author AK facilitated the session by presenting the purpose of the evaluation, discussing the Blackstock framework, and outlining potential criteria for the evaluation which emerged from the framework and preliminary analyses. Illustrative challenges and solutions on each evaluation criteria were presented to the group based on initial data collected through the written reflections and phone calls. Participants were asked to reflect on each criteria and assess if it was appropriate for the consortium’s evaluation. The group responded to proposed challenges and solutions mapped to the criteria and suggested additional examples based on how the criteria applied to their experiences. Notes were taken by two session observers and later compiled.

Based on the notes from the consortium meeting, a Google Doc© was created and shared with each working group member. The document included a table which defined each criteria (see Table 1) and presented illustrative examples of challenges and solutions which emerged from the meeting (see Table 2). Group members were asked to review each criterion, apply it to their setting, and provide additional examples to the table where relevant. These results are presented below.

**Table 1:** List of criteria and definitions

Evaluation Criteria	Definition
Access to resources	Provision of support to allow teams to engage and meet expectations.
Expectation setting	Creation of an agreed and clearly defined vision, objectives, and goals for the project.
Organizational context	Culture, structures/systems, climate, and technology within an institution that influence the project, team members, or partnerships.
External context	Characteristics of the external environment that may influence the project, team members, or partnerships.
Quality of information	Adequacy, quality, and quantity of information provided
Relationship building	Issues of social capital through new and existing social networks developed during the project (e.g. trust, reciprocity, collaboration).
Transparency	Understanding how and why decisions are made; external - where process can be audited
Motivation	Desire and willingness to engage with the project, team, and/or partnership
Scheduling	The timeline and organization of project activities.
Adaptation	The modification of project aspects and partnership engagement.
Communication and engagement	The quality and flow of information within teams and to teams.
Capacity building	Developing relationships and skills to enable teams to take part in future processes or projects.

**Table 2:** Mapped challenges and solutions to each criteria

Evaluation Criteria	Illustrative Challenges	Proposed Solutions
Access to resources	*Financial –* contract and payment delays *Human –* timely recruitment of qualified staff *Technical –* diverse set of unfamiliar online tools; lack of internet connectivity	- Time for recruitment, reflection, and pre-testing. - Technical training. - Permissions to utilize JHU tools (e.g. library access).
Expectation setting	Lack of detailed description of task details. Communication approaches to altered tasks and timelines. Managing expectations of in-country stakeholders.	- Pre-proposal submission meeting with all team members. - Protocol development for communicating adaptations and iterations.
Organizational context	Competing priorities for time and effort. Staff turnover and lengthy hiring processes. Coping capacity with delays.	- Provision of one coordinator for each country team. - Integration of project activities with doctoral program requirements in academic institutions.
External context	Outbreaks (circulating vaccine-derived poliovirus and Ebola) National Elections Ongoing insecurity	- Flexibility in project targets. - Formal communication between JHU and country stakeholders.
Quality of information	Too much vs. insufficient data collection (complexity of processes and tools). Reliability between researchers.	**-** Flexibility in tool modification and deliverable timelines. - More focused and precise tools.
Relationship building	Trust and weak networks between country-team institutions and external stakeholders.	- Leverage JHU name to facilitate networks. - Conduct stakeholder meetings prior to project launch.
Transparency	Lack of early engagement in decision-making for establishing processes, developing tools, and publication.	- Co-developed procedure for data analysis and establishing authorship criteria.
Motivation	Researcher fatigue due to lengthy, detailed processes and tools.	- Rapid publication of results. - Additional time for planning and training.
Scheduling	Frequent timeline adjustment due to holidays, delays, time zones, differing working-day schedules and other external context.	- Regular review (quarterly) of timelines.
Adaptation	Misunderstandings about adapted timelines and work streams.	- Hire additional staff and engage students. - Early planning and engagement with teams on tool and process development.
Communication and engagement	Confusion on who to contact within teams for different streams of work. Onerous communications through different modes.	**-** Additional one-on-one meetings. - Clarity on roles and responsibilities. - Development of externally facing website.
Capacity building	Limited familiarity with diverse set of tools and approaches	- Additional training on tools. - Engaging student researchers. - Increased south-south collaboration.

## Results

Fourteen evaluation criteria emerged from the team’s written reflections, 10 of which are also described by Blackstock et al. No new criteria were established during the calls with consortium member teams. Two evaluation criteria were added during the consortium-wide meeting including capacity building, and social capital and power, the former of which is also included in Blackstock’s framework. During the consortium meeting one criteria, compatibility, was not considered important by the group and was removed from the originally compiled list. Three criteria were combined based on the definitions presented by Blackstock et al. with agreement from the evaluation working group; we merged amount of information with quality of information, engagement with communication, and relationship building with social capital and power. In total, 12 criteria were used to evaluate the STRIPE consortium’s research process and partnership in the project’s first year. These criteria and their definitions are presented in Table 1.

Table 2 outlines each of the evaluation criteria, illustrative challenges from the project, and provides recommended solutions, mapped to those challenges.

### Access to resources

Every team member reflected on this criteria and agreed that it included financial, human, and technical resources. Delays in contracts and payments between the primary and sub-institutions emerged as a common challenge – likely a consequence of working with different administrative systems across institutions. Recruiting human resources qualified to undertake data collection activities was a challenge both at JHU and among partner institutions, especially ones who had large geographical areas to cover during the research, such as in DRC. One team member noted that, “given the complexity of the tasks [STRIPE project], much time was spent in providing constant oversight for quality assurance”. Partner institutions also noted challenges with using online data collection software such as Qualtrics©, both in terms of having inadequate training and capacity for utilization, and the lack of internet in all places, not allowing for active monitoring and analysis of the data. Team members’ recommendations coalesced around the concept of providing more time for recruitment, training, reflection, pre-testing, and financing. Teams also suggested setting up permissions for partner institutions to access the JHU library for literature and other software licenses.

### Expectation setting (shared vision and goals)

Expectations were set and aligned with members of the consortium during the initial phase of the project around work streams, timelines, and the number and scope of project tasks. One team member reflected that, “the vision, objectives and goals were agreed upon upfront, but the devils were in the (technical) details”. Partners noted that communication around alterations in timelines and prioritization of tasks or the addition of new elements was not always effective and resulted in uncertainty and delays. Some teams also reflected on challenges in managing expectations of in-country stakeholders who were expecting immediate benefits of the project such as policy recommendations. A pre-proposal submission meeting was recommended to clarify technical nuances and set realistic targets and timeframes. Setting expectations around adaptations and iterations throughout the process, effective communication styles, and more discussion and clarity in early stages were also suggested.

### Context

The context criteria from the Blackstock framework was differentiated in this research by “organizational” and “external” contexts.

#### Organizational

Each institution and institutional representative faced competing priorities for their time and attention. Staff turnover and lengthy hiring and training processes were two common challenges. The partner institutions also had differing levels of “coping capacity” and “coping strategies”. For example, some institutions were able to cope with delays in financing by pulling from other resources while some institutions did not have other resources available to them and had to delay work and recruitment until all payments were made. Partners suggested hiring and remunerating a minimum of one full-time project coordinator in each setting to manage resources, as well as integrating research projects with PhD programs to avoid high turnover and recoup time spent in hiring and training.

#### External

During the study period DRC faced Ebola outbreaks in many provinces, Afghanistan, Bangladesh, and DRC had elections, Indonesia and DRC experienced the re-emergence of circulating vaccine-derived poliovirus (cVDPV), and insecurity and conflict continued in both Afghanistan and Eastern DRC. These significant events caused competition for stakeholders’ time and attention and made some areas of the country inaccessible for research. In Indonesia the outbreak raised interest in polio and the STRIPE project, but many in the polio universe became more focused on the vaccine-derived poliovirus case rather than other aspects of the study. Teams also reflected on benefits to in-person meetings at times and that they could even be detrimental at other times, depending on the external context. Teams suggested greater flexibility in project targets based on the external context. Additionally, ensuring formal communications from JHU to local governments and core GPEI partners regarding the project, its tasks and timelines could have facilitated greater ownership processes by the partner institutions.

### Quality of information

Reflections on the adequacy, quality, and quantity of information emerged at both the research and partnership level. There were concerns that too much data was collected on some aspects and not enough on others and that there might be limited reliability between reviewers for some arms of the research. The complexity and length of the survey, key informant interviews, and context analysis tools made focusing on the research objective a challenge for data collectors and respondents. Partners also reflected that tools and processes were well-established by JHU early-on and it was unclear how much room existed for feedback and change. Country teams recommended they should be given more flexibility to modify the tools and timelines relevant for the country context and that tools could be more focused and precise, derived from the research questions.

### Relationship Building & Social Capital

Team members first applied these criteria to their relationships with local governments and agencies. Trust between the stakeholders, including government, GPEI partners, and the research institution was raised as a challenge for conducting the project. In some instances, the lack of trust significantly delayed accessing national data. Human resource turnover within Ministries of Health at the national, provincial, and district level negatively influenced team relationship building with key stakeholders. Some teams also described themselves as having “weak networks” and “unable to reach key players”. The power of the institution emerged as a theme. Some teams successfully leveraged (or recommended doing this more often) the name of Johns Hopkins or of the project’s funder. Indonesia particularly noted that some of their stakeholders insisted on national leadership and local ownership, so they had to navigate perceptions of the project being externally driven. Stakeholder engagement prior to data collection was a successful strategy for at least three country teams. The teams in India and Bangladesh conducted stakeholder workshops prior to data collection to introduce the project and facilitate data collection activities. The team in Nigeria conducted advocacy visits to Ministry leadership and heads of agencies involved in polio eradication. Regular communication with stakeholders was widely recommended. The teams then applied the criteria to relationship building within the consortium and reflected on its strength, facilitated by JHU leadership.

### Transparency

This criterion is both internal to team decision making and external to the auditing of decisions and processes. Related to quality of information, transparency concerns emerged around project development (including tools and processes) and expected implementation of project processes. Reflecting on the analysis and write-up phases, country teams said that they would have preferred earlier engagement in decision-making. This included early engagement on decisions around authorship and cross-country analysis plans. Early engagement on tool development, data collection and analysis strategies, and a procedure for defining authorship criteria and processing journal publications and conference presentations was recommended.

### Motivation

Many of the data collection processes for the STRIPE project were detailed and lasted longer than expected. All teams expressed a decrease in motivation during the literature reviews which was especially extensive, particularly with back to back deliverables over a short-time period. The survey and KII tools were both criticized for being lengthy resulting in decreased motivation for participants and researchers. The Health Systems in Transition (HIT) tool, which assessed country contexts over a period of time, was overwhelming for teams to complete due to the volume and nature of the data requested, which required extensive human effort in accessing archives and data not publicly available. While teams did utilize the tool and ultimately saw value in its contributions to context mapping and to the larger project, this laborious task decreased overall motivation. Team members recommended additional time dedicated to project planning and training to align expectations and prepare teams and participants. Some also recommended rapid publication of study results and explicit appreciation to boost researcher motivation.

### Scheduling

The timeline and organization of project activities emerged as a common challenge. Timelines were adapted and frequently changed based on country team needs which made overall project management more difficult. Some team members highlighted the importance of country context and recommended it be taken into consideration when scheduling any research activity; this includes time zones, standard working days, and holidays. Extended holidays around Christmas, New Year, and Eid caused delays in data collection and analysis. Others recommended reviews of these timelines and deliverables with country teams on a quarterly basis.

### Adaptation

Country teams discussed their processes for adapting to shifting timelines and increased workloads. Some hired additional staff and increasingly engaged students. Some teams mistakenly assumed that some aspects of the project replaced others, creating confusion and leading to missed deadlines. Teams noted both success and challenges in having to adapt strategies and schedules due to contextual challenges. The Afghanistan team successfully adapted its approach by employing a phone-based survey, instead of distributing it online, to increase reach and response rates. The DRC team noted challenges with adapting schedules when meetings were planned last-minute. Country teams generally reflected that the JHU team was responsive to requests for adaptation, though recommended a shortening and simplification of the research tools in addition to improved planning of workload roll out.

### Communication & Engagement

Both the country teams and JHU team reflected there was confusion on who to contact for which streams of work and how to communicate when new work streams were being introduced. Mode of communication was also discussed. Teams reflected on receiving too many emails with too much information while also not being able to attend monthly calls due to time differences and scheduling conflicts. Teams also noted challenges in keeping up to date on tasks and products across partners due to the lack of common communication platforms that could be used by all. Additional one-on-one meetings were suggested, both via phone and in-person. Teams also recommended clarity on the structure of the JHU team, outlining roles and responsibilities. External communication with country stakeholders was also viewed as a challenge for some. The working group agreed that an externally facing project website would help improve communication across teams and with stakeholders about the purpose and findings of the project.

### Capacity building

The knowledge mapping phase of the project was multifaceted, asking teams to complete a variety of tasks requiring different types of expertise. Team members mentioned that they had limited familiarity with the tools (e.g. Qualtrics©, an online survey tool, and F1000, an online literature sharing and storing tool) and processes (e.g. transcription, memoing, and scoping review analysis). Additional training on the tools, analysis, and writing were recommended. Engaging student researchers was a valuable strategy; the vast amount of data collected by each team provided opportunities for developing dissertation projects based on field work. Teams recommended more south-south collaboration, learning from each other’s strengths. Integrated teams at JHU from different disciplines built capacity within the team; that is, qualitative and quantitative researchers learned from each other’s approaches and methodologies and gained better understanding of the mixed-methods research process as a whole.

## Discussion

This paper reflects on the collaborative and research process of academic and research institutions in eight different countries. The experience from participation within the consortium indicates that successful management of multi-country, multicentered implementation research requires comprehensive communication tools, expectation setting, and institutional support.

Clear and transparent communication is needed throughout the research process to establish relationships and maintain momentum in the project [12]. In this era of advanced technology there remains a dearth of project management platforms that can be easily understood, accepted, and utilized in different settings [13]. Throughout the first year of STRIPE different tools were introduced including Zoom©, Google Docs©, F1000©, Qualtrics©, and Dropbox© but these were insufficient and disconnected. Partners with unstable internet were less able to utilize complex systems requiring bandwidth; others struggled to easily locate project materials. Simple project management tools that are responsive to the needs and calendars of diverse contexts are needed. This should include an online/offline syncing capability, document sharing with easy editing and commenting, and a calendar/scheduling tool. While some of these tools exist, institutional processes often do not allow us to share and use the platforms with external partners. The introduction of existing tools (or future new tools) must be accompanied by training so all partners are familiar with features and buy-in to the approach.

How collaboration is started, its early processes, and relationship management are arguably at as least important as the strength of the collaboration’s strategic premise [14, 15]. Therefore, buy-in from all partners is needed in early stages of any project to align expectations [12]. While an initial consortium meeting was held prior to the launch of any research activities, more preparation and clarity was needed by partners regarding the mission, vision, goal, and scope of work. Some of this was accomplished by hosting a single meeting of the partners but there was a tension between not wanting to be prescriptive (that is, not having a top-down approach led by JHU) and needing to direct. This meeting also facilitated project ownership by subcontracted country teams that provided substantial input into the tools and processes that would be used. Start-up meetings must address expectations for activities, document sharing (from concept notes and drafts to finish products), timelines (including adaptations), collaborative opportunities (when feedback is wanted, needed, and/or welcome), and clear communication strategies.

Expectation alignment also prepares institutions for their human resource needs [12]. The development of clear scopes of work facilitates the budgeting and hiring of qualified, well-trained personnel who can dedicate the required time to completing project tasks. Faced with increasing workloads many partners filled gaps within their teams through rapid hiring processes. Institutional requirements in hiring delayed timelines for some teams and increased the work burden on core personnel. Institutions differed in their capacity to withstand delays (e.g. arrival of financing) and cope with changing demands. Consortia should assess each member’s coping capacity and develop strategies to support institutional adaptations. Capacity building activities within a project should focus not only on the technical skills of the individuals, but also on the institution’s structures and systems. This approach requires significant resources, both human and financial. For example, well-staffed departments dedicated to providing information technology, regulatory management, and external communications and outreach, could facilitate more than one project and increase productivity of the research team. Funding institutions have been criticized for not emphasizing capacity building in their proposal requirements or allotting appropriate resources for capacity building activities. For consortia grantees to achieve this recommendation, funding institutions must require and allocate resources for organizational-level capacity building.

As briefly stated earlier, there is an important limitation to this partnership process evaluation, namely that it was conducted among subcontracted partners with involvement from the lead organization (JHU). Feedback from country team participants was likely influenced by a desire to maintain professional and interpersonal relationships with other teams, including JHU. We therefore recognize that power dynamics may have influenced the data itself and the processes of data collection and analysis. To mitigate these concerns, other consortia seeking to conduct such an evaluation could consider hiring an independent third-party evaluator. Consortia would likely need to plan and budget for this when applying for initial funds. Our evaluation also relied on shared experiences (i.e. the working group meeting) and shared tools (i.e. Google Docs) to capture cross-country themes and illustrative examples. While we believe this approach facilitated openness and group-learning, team members may have withheld important information. Future evaluations could emphasize data collection at the individual or team level using group consensus approaches building later in the process.

## Conclusion

This collaboratively conducted partnership process evaluation highlights the importance of clear and open communication, early expectation alignment, and the development of capacity building activities that address human resource needs for individuals and their institutions. Improved collaborative project management tools are needed for academic consortia to effectively communicate about the partnership and research processes. Multiyear academic consortia should conduct such evaluations which can provide useful insights for the consortia itself and to those seeking to build and maintain their own cross-country partnerships.

## Data Availability

Data and publications from this project will be open access and available via an online repository.

## Abbreviations

AIRP-SD: Adaptive Integration of Research and Policy for Sustainable Development
CIOMS: The Council for International Organizations of Medical Sciences
cVDPV: Circulating vaccine-derived poliovirus
HiT: Health Systems in Transition
JHU: Johns Hopkins University
KII: Key Informant Interviews
LMIC: Low- and Middle-Income Country
STRIPE: Synthesis and Translation of Research and Innovations from Polio Eradication
WHO: World Health Organization
